# Biocompatible Aloe vera and Tetracycline Hydrochloride Loaded Hybrid Nanofibrous Scaffolds for Skin Tissue Engineering

**DOI:** 10.3390/ijms20205174

**Published:** 2019-10-18

**Authors:** Hariharan Ezhilarasu, Raghavendra Ramalingam, Chetna Dhand, Rajamani Lakshminarayanan, Asif Sadiq, Chinnasamy Gandhimathi, Seeram Ramakrishna, Boon Huat Bay, Jayarama Reddy Venugopal, Dinesh Kumar Srinivasan

**Affiliations:** 1 Department of Anatomy, Yong Loo Lin School of Medicine, National University of Singapore, Singapore 117594, Singapore; anthe@nus.edu.sg (H.E.); antbaybh@nus.edu.sg (B.H.B.); 2 Department of Mechanical Engineering, Center for Nanofibers & Nanotechnology, Faculty of Engineering, National University of Singapore, Singapore 117576, Singapore; raghavendra8025@gmail.com (R.R.); drancer.asif@gmail.com (A.S.); seeram@nus.edu.sg (S.R.); jrvgopal@gmail.com (J.R.V.); 3 Anti-Infectives Research Group, Singapore Eye Research Institute, Singapore 169856, Singapore; Chetnachem24@gmail.com (C.D.); lakshminarayanan.rajamani@seri.com.sg (R.L.); 4 Temasek Life Sciences Laboratory, National University of Singapore, Singapore 117604, Singapore; gandhi@tll.org.sg; 5 Faculty of Industrial Sciences & Technology, Universiti Malaysia Pahang, Gambang 26300, Malaysia

**Keywords:** electrospinning, nanofibers, curcumin, aloe vera, tetracycline hydrochloride, fibroblasts, wound healing

## Abstract

Aloe vera (AV) and tetracycline hydrochloride (TCH) exhibit significant properties such as anti-inflammatory, antioxidant and anti-bacterial activities to facilitate skin tissue engineering. The present study aims to develop poly-ε-caprolactone (PCL)/ AV containing curcumin (CUR), and TCH loaded hybrid nanofibrous scaffolds to validate the synergistic effect on the fibroblast proliferation and antimicrobial activity against Gram-positive and Gram-negative bacteria for wound healing. PCL/AV, PCL/CUR, PCL/AV/CUR and PCL/AV/TCH hybrid nanofibrous mats were fabricated using an electrospinning technique and were characterized for surface morphology, the successful incorporation of active compounds, hydrophilicity and the mechanical property of nanofibers. SEM revealed that there was a decrease in the fiber diameter (ranging from 360 to 770 nm) upon the addition of AV, CUR and TCH in PCL nanofibers, which were randomly oriented with bead free morphology. FTIR spectra of various electrospun samples confirmed the successful incorporation of AV, CUR and TCH into the PCL nanofibers. The fabricated nanofibrous scaffolds possessed mechanical properties within the range of human skin. The biocompatibility of electrospun nanofibrous scaffolds were evaluated on primary human dermal fibroblasts (hDF) by MTS assay, CMFDA, Sirius red and F-actin stainings. The results showed that the fabricated PCL/AV/CUR and PCL/AV/TCH nanofibrous scaffolds were non-toxic and had the potential for wound healing applications. The disc diffusion assay confirmed that the electrospun nanofibrous scaffolds possessed antibacterial activity and provided an effective wound dressing for skin tissue engineering.

## 1. Introduction

The skin is a critical structure comprising of an epidermis (which acts as a waterproof layer) followed by the dermis which gives the ability to withstand wear and tear [1]. As the largest organ in the body of human, covering approximately 8% of total body weight, with a thickness that varies between 1.5 to 4.0 mm, the skin acts as a barrier to distinguish exterior from interior surroundings. The loss of skin tissue by injuries, such as lacerations and burns (>4 cm thick), lead to an inadequate healing process. Surgical treatment by conventional methods, such as autografts and allografts, may be the only solution for chronic wounds. However, these methods have limitations that include the morbidity of donor sites, transmission of infections and immune-rejection [2]. An effective wound healing process could include cell to cell interaction, vascularization and extracellular matrix (ECM) secretion. The ECM comprises of collagen, elastin growth factors, bioactive molecules and fibronectin, all of which are controlled via cell signaling by cytokines and hormonal proteins that help in determining the characteristics of the cells, such as proliferation, adhesion and migration [3].

Regenerative medicine is a promising field that encompasses a tissue engineering approach by combining life sciences with engineering that helps in developing biological substitutes to repair, retain and expand the functions of tissue, through understanding the behavior of normal and diseased or injured tissues. A modern approach of tissue engineering which has paved the way for the healing of chronic wounds is fabrication of skin substitutes [4]. Inchingolo et al. fabricated a grafting material with platelet rich plasma in which the in-vitro study demonstrated that fabricated material actively aided in bone tissue regeneration [5]. A 3D tissue engineering construct of sericin using the co-culture of keratinocytes and fibroblast, has demonstrated the significance of paracrine signaling between keratinocytes and fibroblast in the expression of the ECM protein for dermal repair [6]. Tissue engineering works by combining cells and scaffold which mimics the ECM [7]. A cell-scaffold interaction induces signaling for cell behavior making the ECM composition a crucial factor in proliferation, growth and differentiation. The physiology of the ECM is a connective network composed of fibrous glycoprotein that co-ordinates in-vivo tissue functions to provide the mechanical stability and biochemical cues necessary for tissue morphogenesis and homeostasis [8]. The ultimate aim of skin tissue engineering is to fabricate skin substitutes so as to accelerate the wound healing process using the principles of: mimicking native physiological skin; guarding the loss of fluid and growth factors; being biocompatible; evading the invasion of microorganisms; promoting the release of cytokines and growth factors at the wound site for skin regeneration [9]. Wound dressings have been fabricated in several structural forms such as nanofibers, films and meshes. The key factors for successful wound repair with the support of biomaterials are based on surface topography, the type of polymer used, fabrication techniques, biocompatibility and biodegradability of the scaffolds [10,11]. 

Electrospinning is a well-known technique for its versatility to fabricate fibers of micro and nanometer scale. Electrospun nanofibers have been used as scaffolds and architectural resemblance to the ECM [12]. Synthetic polymers have good flexibility in fabricating and altering scaffolds, but synthetic compound lacks cell attachment due to their low hydrophilicity and lack of surface cell recognition sites. Compared with synthetic compounds, natural compounds provide good biocompatibility but tend to exhibit poor synthesizing capability and mechanical properties. Therefore, it is advantageous to fabricate composite fibrous scaffolds containing both synthetic polymers for the backbone and natural polymers for cellular attachment, which does not only acquire appropriate mechanical properties, but also a bioactive surface [13,14].

Poly-ε-caprolactone (PCL), is a Food and Drug Administration (FDA) approved polymer renowned in properties such as biodegradability, biocompatibility, chemical stability, thermal stability, good mechanical properties, tissue-compatible nature and permeability [15]. The absence of a cell adhering site, makes the cells difficult to attach for the tissue regeneration. To overcome this limitation, natural components are blended with PCL to produce functionalized nanofibers for wound dressing. Aloe vera (AV) has numerous physiologically active components and possesses antioxidant properties and increases collagen, hyaluronic acid and dermatan sulfate production which are key constituents of the ECM that lead to effectual wound healing. Aloe vera is particularly appealing in the field of regenerative medicine as it supports cell migration, proliferation, and growth [16,17]. Curcumin (CUR), an active component of turmeric, is well-known to display properties such as antioxidant, anti-inflammatory, antitumor, anticoagulant and antimicrobial activity which are favorable factors for wound healing [18,19]. Tetracycline, is an antibiotic with antimicrobial and anti-inflammatory properties [20]. Soares et al. have proposed that the treatment of tetracycline hydrochloride (TCH) in dentin could increase the fibroblast attachment and growth by binding to the ECM glycoprotein and fibronectin [21]. The incorporation of TCH may support the regeneration of fibroblasts for an effective wound healing by its controlled release from the nanofibrous scaffolds. Anges et al. reported that PCL/AV supports fibroblast cell proliferation without the cytotoxic effect for skin tissue engineering [22]. Mesenchymal stem cells (MSCs) popularly differentiate into many descents such as cartilage, bone, muscles and adipose tissues. MSCs stimulate wound repair by increasing angiogenesis, promoting resolution of wound inflammation, favourably regulating the ECM remodelling, and driving the regeneration of skin with normal architecture and functions [23]. Adipose derived stem cells (ASCs) play an indispensable role in the repair of skin wounds more than stem cells because of their advantages, such as immune compatibility and freedom from ethical concerns. Despite its advantages, clinical trials on the efficacy of ASCs are very few and only in the early stages. More clinical trials are still required to verify the therapeutic value of ASCs in wound repair [24]. The objective of this study is to analyze PCL and AV loaded with CUR and TCH hybrid nanofibrous scaffolds for cell cytotoxicity, the secretion of collagen and antimicrobial activity for the application of wound healing in skin tissue engineering. 

## 2. Results and Discussion

### 2.1. Characterization of Nanofibers

**Figure 1:**
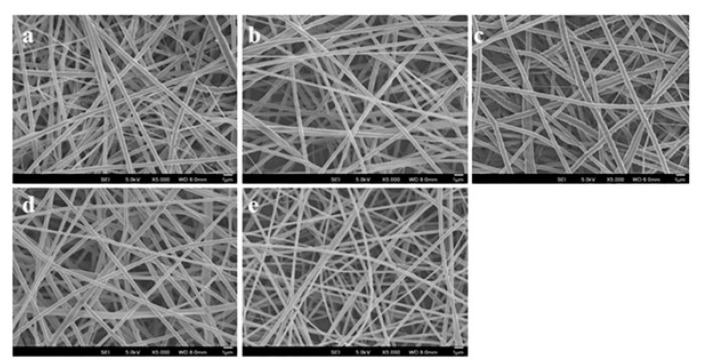
SEM images for biocomposite nanofibrous scaffolds (**a**) poly-ε-caprolactone (PCL), (**b**) PCL/ aloe vera (AV), (**c**) PCL/ curcumin (CUR), (**d**) PCL/AV/CUR (**e**) PCL/AV/ tetracycline hydrochloride (TCH). Scale bar = 1µm.

The development of the biocompatible wound dressing nanofiber mesh is highly dependent on the characteristic properties of the nanofibers. Electrospun nanofibers can be fabricated in a nanoscale dimension by both synthetic and natural polymers to mimic the native physiological ECM. It has characteristic features, such as high porosity, biocompatibility, well interconnected pores, and a large surface area which promotes cell growth, adhesion and spreading along the nanofibers for enhanced wound healing. The cell adhesion, proliferation and immobilized release of biomolecules are influenced by the morphology and internal structure of the nanofibers [25]. Electrospun nanofibers were analyzed under a field emission scanning electron microscope (FESEM) at an accelerating voltage of 15 kV. FESEM is a useful technique to observe the basic characteristics such as the morphology, size, shape of nanofibers. Figure 1 displays the surface morphology of nanofibers PCL, PCL/AV, PCL/CUR, PCL/AV/CUR, and PCL/AV/TCH analyzed by FESEM. All nanofibers revealed randomly oriented bead free, smooth surfaced morphology with a uniform distribution of nanofibers.

The average fiber diameter of PCL, PCL/AV, PCL/CUR, PCL/AV/CUR, and PCL/AV/TCH scaffolds were 770 ± 98 nm, 561 ± 49 nm, 695 ± 57nm, 665 ± 64 nm and 360 ± 87 nm respectively. Among all the scaffolds, PCL (770 ± 98 nm) displayed higher mean fiber diameter while PCL/AV/TCH (360 ± 87 nm) showed a lower fiber diameter indicating a decrease in the average fiber diameter (Table 1). The significant variations in the diameter of the scaffold were observed due to the incorporation of bioactive components. There was a decrease in fiber diameter upon blending with natural polymer AV (561 ± 49 nm) and the nanofiber diameter decreased further with the incorporation of TCH into the PCL/AV hybrid structures. The decrease in fiber diameter could be possibly due to the increase in conductivity of polymer solution by the addition of biological substitutes (AV and TCH) [26]. The incorporation of CUR to PCL (695 ± 57 nm) and PCL/AV (665 ± 64 nm) showed an increase in the fiber diameter which may be due to the precipitation of CUR in nanofibers as reported by Shababdoust et al. [27].

The affinity of the cells towards the surface of the biomaterial is a key consideration for the fabrication of a successful skin substitute. The hydrophilicity of nanofibers is a primary criterion for the cell adhesion which leads to cell spreading and focal adhesion along the nanofibers for cell proliferation [28]. The surface wettability and porosity of the nanofibrous scaffolds supports the cells in maintaining a constant supply of nutrient and excretion of cellular waste by diffusion. The water contact angle which is a basic technique that determines the surface wettability of mats by a liquid [26,29]. Table 1 shows the water contact angle values of the electrospun mats in which the values greater than 90° delivers poor spreading of liquid over the nanofibers while values less than 90° resemble the characteristics of the water absorbing surface (hydrophilic). The contact angle of PCL, PCL/AV, PCL/CUR, PCL/AV/CUR and PCL/AV/TCH were 128.3 ± 6°, 47.3 ± 2.5°, 94.3 ± 3.7°, 79 ± 1.6° and 57.3 ± 5°, respectively. PCL exhibits the water contact angle of 128.3 ± 6° which is above 90°, thus it has hydrophobic properties. The increase in the wettability of PCL mats was observed on adding AV, CUR and TCH. PCL/CUR showed 94.3 ± 3.7° (a decrease in wettability when compared to the PCL/AV nanofibers) which may be due to the hydrophobic nature of CUR [30]. PCL/AV/CUR showed an increase in wettability (79 ± 1.6°) when compared to PCL/CUR (94.3 ± 3.7°), which could be possibly due to the presence of biologically active components in AV. The PCL/AV/TCH nanofibers contact angle showed 57.3 ± 5° to increase the surface wettability when compared to PCL, PCL/CUR and PCL/AV/CUR.

**Figure 2:**
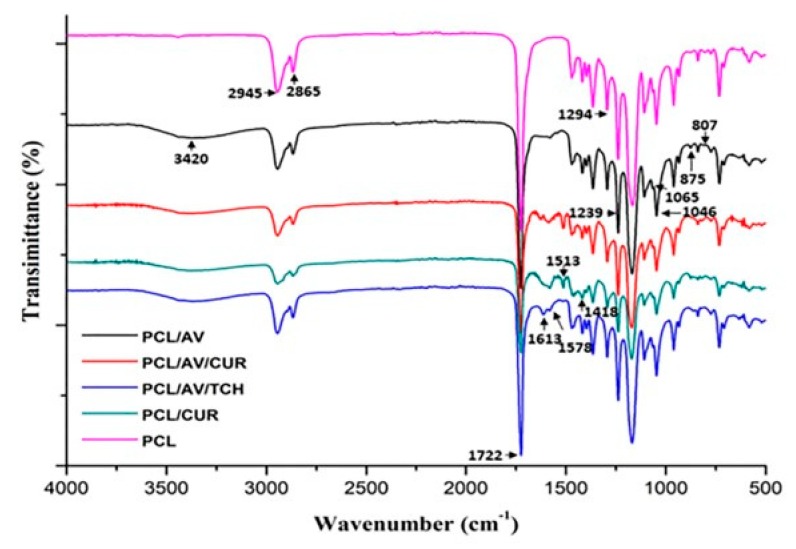
FTIR spectra of biocomposite nanofibrous scaffolds of PCL, PCL/AV, PCL/CUR, PCL/AV/CUR and PCL/AV/TCH.

The functional groups and chemical bonds of compounds incorporated into the nanofibers were analyzed using FTIR spectrum. Figure 2 represents the FTIR spectrum of the various electrospun scaffolds PCL, PCL/AV, PCL/CUR, PCL/AV/CUR and PCL/AV/TCH. PCL showed the characteristic peaks of –CH_2_ (symmetric) and –CH_2_ (asymmetric) vibrations at 2865 cm^−1^ and 2945 cm^−1^ and a predominant peak at 1722 cm^−1^ corresponding to ester stretching which were observed in all electrospun mats to prove the existence of PCL nanofibers. The presence of hydroxyl group at 3420 cm^−1^ in all scaffold except PCL confirms the incorporation of AV, which further the peaks at 800 cm^−1^ to 1000 cm^−1^ providing evidence in the presence of mannose, pyranose, glucan, monopyranose in AV [26]. The stretching vibration at 1513 cm^−1^ (C=C) and bending vibration at 1418 cm^−1^ (C-H) correspond to the characteristic peaks of CUR [31]. PCL/AV/TCH nanofibers show peaks at 1613 cm^−1^ (C=O A and C ring) and 1579 cm^−1^ (NH_2_ amide), which correlate with the presence of TCH [32].

**Figure 3:**
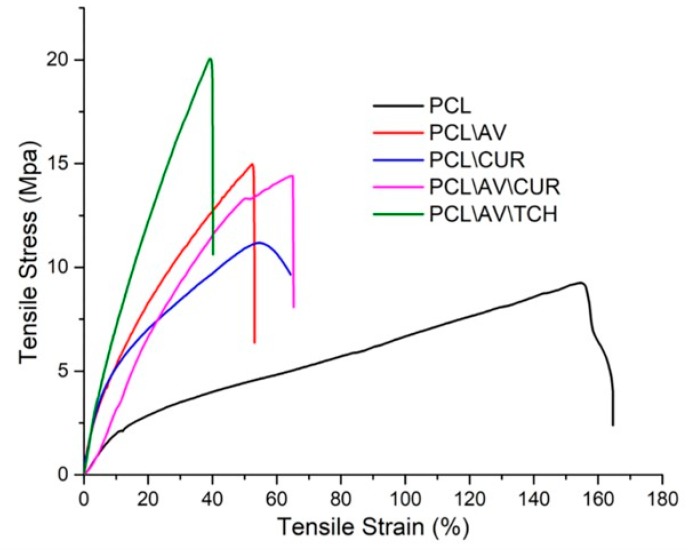
Tensile properties of PCL, PCL/AV, PCL/CUR, PCL/AV/CUR and PCL/AV/TCH nanofibrous scaffolds.

The mechanical properties of nanofibers are critical factors for skin tissue engineering in order to withstand the physiological forces, such as a substantial vascular network, nerve bundles, collagen deposition and other skin anatomy in the wound healing process. Figure 3 and Table 2 show the non-linear graph of the stress-strain curve and the values of ultimate tensile stress, ultimate tensile strain and Young’s modulus of various electrospun samples. PCL displayed tensile strain of 9.2 MPa with the maximum elasticity of 164.5%. The stiffness of the PCL mat increased upon the incorporation of AV. The study of Suganya et al. showed that the mechanical strength of the scaffold increases upon the incorporation of AV in the PCL mats [33]. Lee et al. proposed that increasing the crystallinity of nanofibers by the freezing and thawing technique, made it prime to elevate the mechanical strength of the nanofibers [34]. It was observed, in this study, that PCL/AV/TCH has a maximum stress of 20 MPa (Figure 3), which may be due to the increase in crystallinity of the nanofibers upon the incorporation of crystalline TCH into the PCL/AV nanofibers. Among all the samples, PCL/AV/TCH (360 ± 87 nm) recorded a smaller fiber diameter, while PCL/CUR showed a higher fiber diameter of 695 ± 57 nm. Hence, it can be inferred that a rise in the ultimate tensile strength value of nanofibers is directly proportional to a decrease in the fiber diameter i.e., by increasing the packing density of the fibers by decreasing the fiber diameter primes the increase of the tensile strength [35].

### 2.2. Drug Release

**Figure 4:**
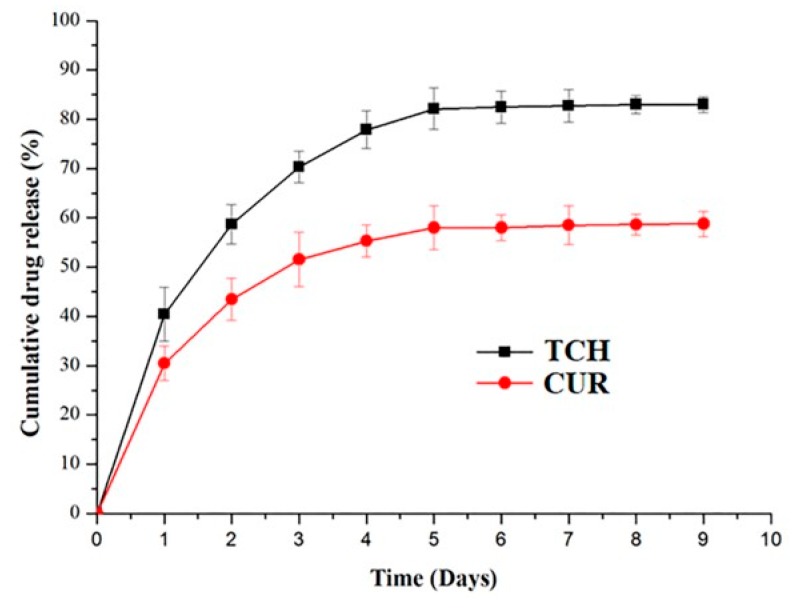
Drug release profile kinetics of PCL/AV/CUR and PCL/AV/TCH nanofibers.

The release of CUR and TCH were analyzed by immersing 50 mg of PCL/AV/CUR and PCL/AV/TCH mats in 3 mL PBS solution (37 °C, pH 7.4). Figure 4 represents the uniform release kinetics of CUR and TCH for a period of 9 days, with the initial burst release of 30.1% and 41.5%, respectively. This is followed by the sustained slow release of drugs which is present in the core structure of the nanofibers. The burst release occurrence could not be avoided as the drug (CUR and TCH) were co-electrospun with polymers and immobilized at the surface area of the nanofibers [36]. Chen et al. reported that the scaffold with a lower fiber diameter displayed a higher release of the drug compared to the scaffold with a larger diameter as the drug could not diffuse faster from the interior of the larger diameter nanofibers [37]. Similarly, due to higher specific surface area from the lower diameter nanofibers, PCL/AV/TCH with 360 ± 87 nm displayed a higher release of drugs when compared to PCL/AV/CUR with 665 ± 64 nm in diameter. The sustained slow release was followed by a linear release behavior of PCL/AV/CUR and PCL/AV/TCH until day 9. After 9 days of the drug release study 42.1% CUR and 16.9% TCH remained in the nanofibers.

### 2.3. Cell Viability

**Figure 5:**
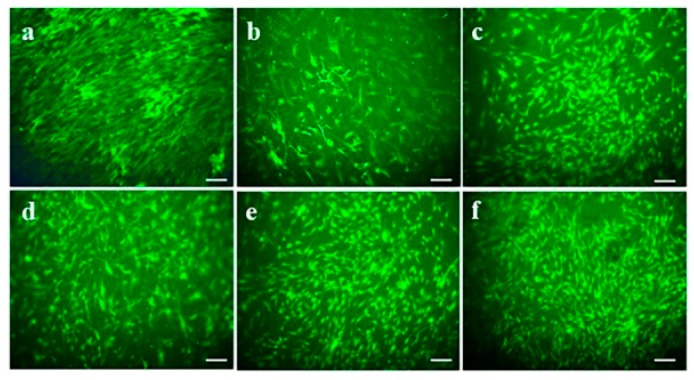
CMFDA live cell imaging of fibroblast on biocomposite nanofibrous scaffolds on day 6 (**a**) TCP, (**b**) PCL, (**c**) PCL/AV, (**d**) PCL/CUR, (**e**) PCL/AV/CUR (**f**) PCL/AV/TCH. Scale Bar = 50 µm.

When the fibroblasts are seeded on biomaterials, the interaction with the scaffolds may disturb the viability of the cells due to the cytotoxic effect of the substances included in the scaffolds. To analyze the effect of TCH and CUR on cell viability of fabricated nanofibers, 5-Chloromethylfluorescein diacetate (CMFDA) assay was performed for the viable cells. CMFDA is cell-tracker dye which provides sharp fluorescence to observe the size, shape and morphology of the viable cells in vitro. The CMFDA compound penetrates through the cell membrane of the viable cells to exhibit a fluoresce acting upon cytosolic esterase enzymes. Figure 5 shows the CMFDA tagged hDFs on day 6 of post seeding in the nanofibers. It was found that fabricated scaffolds were biocompatible and non-toxic to cells. The difference in morphological features was observed between PCL scaffolds and the scaffold loaded with natural polymers such as AV, CUR and TCH. Some of the cells on PCL/CUR displayed structural abnormalities. The lower cell viability observed in PCL and PCL/CUR nanofibers due to the hydrophobic nature of PCL and CUR. In PCL/AV, PCL/AV/CUR and PCL/AV/TCH scaffolds, the cells were distributed around the scaffolds with a normal spindle shaped morphology [38]. PCL/AV/TCH displayed an increased number of cells with better cell to cell interactions and a controlled arrangement along the nanofibers when compared to PCL/AV/CUR. The synergetic effect of AV and TCH could be the possible reason for the observation of the high cell viability in PCL/AV/TCH nanofibrous scaffolds (Figure 5f).

### 2.4. Cell Proliferation

**Figure 6:**
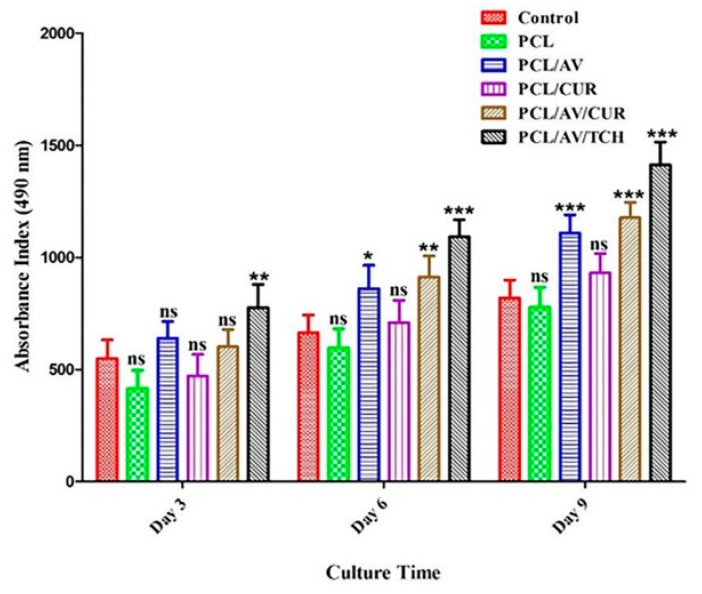
Proliferation of fibroblast on biocomposite nanofibrous scaffolds on day 3, 6, 9 in TCP, PCL, PCL/AV, PCL/CUR, PCL/AV/CUR and PCL/AV/TCH. ns: not significant, * *p* < 0.05, ** *p* < 0.01, *** *p* < 0.001.

For the proliferative phase of wound repair, the fibroblasts proliferate to form new tissue granulation composed of procollagen, elastin, proteoglycans and hyaluronic acid (HA). Fibroblasts further differentiate into myofibroblasts with enhanced α-smooth muscle actin cytoskeleton which aids to wound contraction and the ECM formation [39]. To analyze the biocompatibility and cell proliferation potency of nanofibers, MTS proliferation assay was performed. Figure 6 shows the hDF proliferation on the tissue culture plate (TCP), PCL, PCL/AV, PCL/CUR, PCL/AV/CUR and PCL/AV/TCH nanofibers on day 3, 6, and 9. PCL shows a lower proliferation rate when compared with all other scaffolds which could be possibly due to the absence of the cell recognition motif in PCL that fails to form focal adhesion between the nanofibers and the seeded cells. On day 3, 6, and 9, PCL/CUR displayed lower proliferation when compared to PCL/AV, PCL/AV/TCH and PCL/AV/CUR as CUR possesses a hydrophobic character which may have disturbed the fibroblast adhesion on the scaffolds. However, PCL/AV/CUR showed increased proliferation when compared to PCL, PCL/AV and PCL/CUR which could be due to the release of hydrophobic drug CUR by the augmentation of the hydrophilic character of nanofibers through the immobilization of AV. Nina et al., who have studied TCH as a model drug in PCL/CA/Dextran/TCH nanofibrous scaffolds, observed better antibacterial and antifungal activities with improved fibroblast proliferation in scaffolds containing TCH [32]. It is evident that PCL/AV/TCH nanofibers facilitate the high proliferation of hDF compared with all the other scaffolds (Figure 6). The proliferation results suggest that the drug loaded scaffold was nontoxic and biocompatible to stimulate the proliferation of dermal fibroblasts for enhanced wound healing.

### 2.5. Expression of Collagen

**Figure 7:**
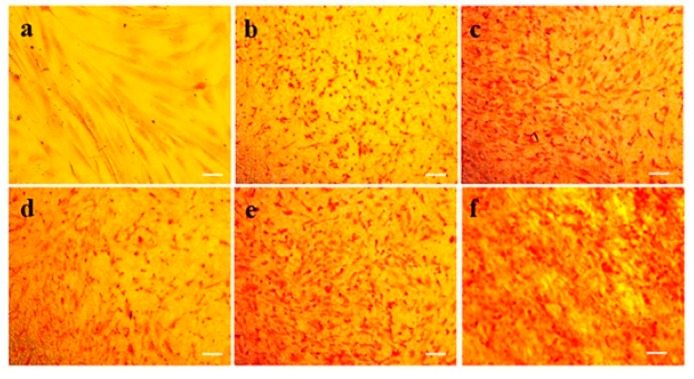
Extracellular matrix (ECM) deposition of fibroblasts by Sirius red staining for collagen expression on Day 6 (**a**) TCP, (**b**) PCL, (**c**) PCL/AV, (**d**) PCL/CUR, (**e**) PCL/AV/CUR, (**f**) PCL/AV/TCH. Scale Bar = 50 µm.

Collagen is a primary component of ECM and also an abundant protein in mammals. Collagen has a stiff triple-helical structure of a three molecular chain which supports the ECM for high stiffness and has anisotropic mechanical properties [40]. Collagen acts as a structural support for skin and helps in functioning the cell migration, maintaining the cell shape and inducing protein synthesis. For the proliferative phase, fibroblasts produce collagen which replaces the fibronectin-fibrin matrix that produce structure to the wound. In the remodeling phase, myofibroblasts deposit the collagen by cross linking in the wound which helps in wound contraction along with the increase in mechanical strength of the wound [41,42]. Figure 7 shows the collagen expressed by hDFs seeded on various scaffolds, PCL, PCL/AV, PCL/AV/CUR and PCL/AV/TCH by Sirius red staining on day 6. The reduced level of collagen secretion with structural abnormalities in PCL and PCL/CUR scaffold was clearly observed at different time points. This may be due to the hydrophobic nature of the PCL and PCL/CUR nanofibers. Among all the scaffolds, PCL/AV/TCH displayed a higher distribution of collagen along the nanofibers with an increased intensity (red colour), signifying that PCL/AV/TCH nanofibrous mats supported the enhanced proliferation of hDFs. The increased collagen secretion in PCL/AV/TCH may also be attributed to the presence of TCH, which has the ability to inhibit collagenase activity and elevate the deposition of the ECM proteins with increased fibroblast proliferation [21,43].

### 2.6. Cell-scaffold Interactions

**Figure 8:**
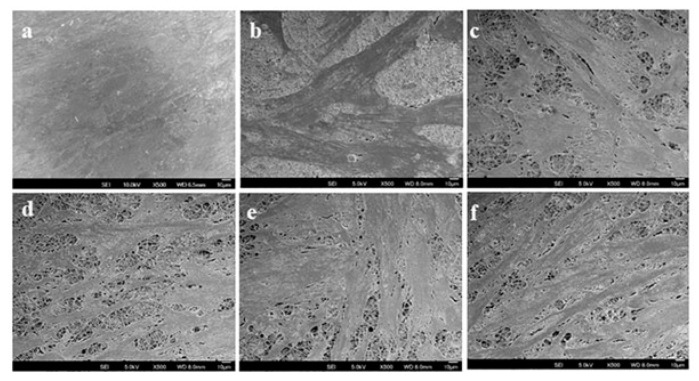
SEM images of fibroblast on the biocomposite nanofibrous scaffolds on day 9 (**a**) TCP, (**b**) PCL, (**c**) PCL/AV, (**d**) PCL/CUR, (**e**) PCL/AV/CUR, (**f**) PCL/AV/TCH.

The physical and chemical property of the fabricated scaffold is responsible for the cells-scaffold interaction, cell communication, nutrients uptake of the cells from the scaffold, molecular cell signaling and the deposition of the ECM components which prime the development of a successful skin substitute [25]. For the effects of the fabricated nanofibers over the cell morphology, cell spreading, cell attachment, a SEM analysis was conducted on the mats incubated with hDFs. Figure 8 shows the SEM images of hDFs cultured on PCL, PCL/AV, PCL/CUR, PCL/AV/CUR and PCL/AV/TCH nano-scaffolds on day 9. For the PCL scaffolds, the cells decreased in number with an altered shape and structure owing to the absence of active integrin sites. The scaffolds with the incorporation of AV showed an increased cell density and cell spreading along the nanofibers which may be due to the presence of polar phytochemicals in AV. The micro-architecture of human connective tissues is dictated by the controlled cellular arrangement which regulates the biological and mechanical function of the tissue. It is important that the tissue engineered constructs the supports for the controlled cell alignment along the nanofibers [44]. PCL/AV/TCH shows improved cell spreading, cell to cell communication and controlled extension of fibroblasts along the nanofibers in comparison to PCL, PCL/AV, PCL/CUR and PCL/AV/CUR nanofibers. Overall, PCL/AV/TCH nanofibrous mats as a wound dressing can support cell attachment, which spreads and promotes proliferation with controlled cell growth for wound healing.

### 2.7. Expression of F-actin

**Figure 9:**
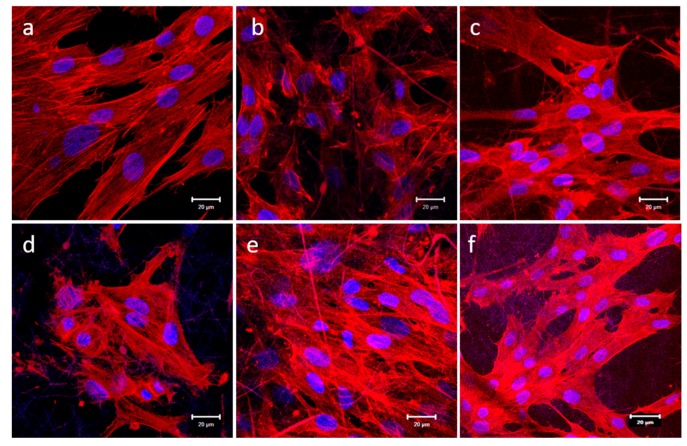
Immunofluorescence analysis of fibroblast expression of specific marker protein F-actin (red) on Day 9 (**a**) TCP, (**b**) PCL, (**c**) PCL/AV, (**d**) PCL/CUR, (**e**) PCL/AV/CUR (**f**) PCL/AV/TCH. Scale bar = 20 µm.

The actin cytoskeleton network subjects for wound contraction help in tissue granulation which enables wound closure [43]. hDFs cultured for 9 days were stained with Rhodamine phalloidin and DAPI. Cytoplasmic F-actin inter-network bundles were stained red by phalloidin and nucleus stained blue by DAPI (Figure 9). PCL and PCL/CUR scaffolds, the distribution of F-actin was not clearly observed due to spatial scattering of F-actin with abnormal cell cytoskeleton morphology. This may be due to the nanofibers hydrophobic effect which can affect the cell cytoskeleton structure by not supporting the cell attachment and spreading. hDFs grown on PCL/AV, PCL/AV/CUR and PCL/AV/TCH displayed an elongated spindle shaped morphology and a notable cytoskeleton structure with the distribution of F-actin fibers throughout the cell cytoplasm when compared to PCL and PCL/CUR, due to the presence of the bioactive components. hDFs on PCL/AV/TCH scaffold expressed a highly aligned F-actin filament through the cell cytoskeleton with a notable elongated fibroblast morphology and better cell to cell interactions when compared to PCL, PCL/CUR, PCL/AV and, PCL/AV/CUR nanofibers.

### 2.8. Antimicrobial Activity

**Figure 10:**
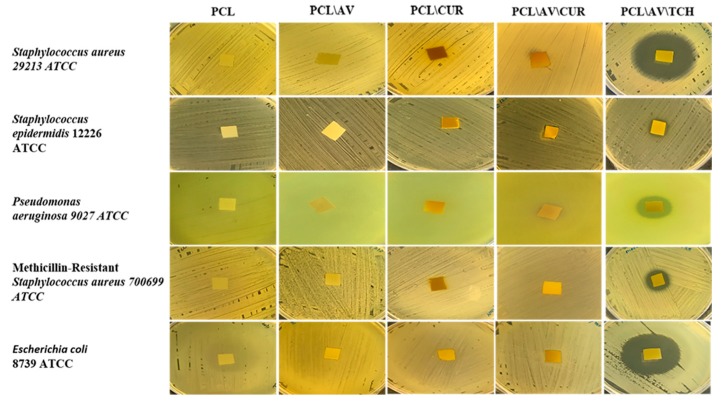
Antimicrobial efficacy of biocomposite nanofibrous scaffolds PCL, PCL/AV, PCL/CUR, PCL/AV/CUR and PCL/AV/TCH.

The antimicrobial efficacy of electrospun nanofibrous scaffolds PCL, PCL/AV, PCL/CUR, PCL/AV/CUR and PCL/AV/TCH were accessed by a Kirby-Bauer disc diffusion assay (Figure 10). The contact mediated inhibition of all five bacteria was noticed, while no clear zone of inhibition was observed around the following mats namely, PCL/AV, PCL/CUR and PCL/AV/CUR mats. No zone of inhibition and contact inhibition was observed in PCL as it is a synthetic polymer without any biologically active properties. In contrast, PCL/AV/TCH represent clear zones around the mats compared to all other nanofibers. The observed results proved that the antibiotic activity of TCH was not disturbed by encapsulating the nanofibrous scaffolds for cell proliferation and the secretion of collagen for wound healing.

## 3. Materials and Methods

### 3.1. Materials

The human dermal fibroblasts (hDF), foetal bovine serum (FBS), Dulbecco’s modified eagle’s medium (DMEM) were obtained from Gibco^®^, Thermo Fisher Scientific, Singapore, Singapore. penicillin-streptomycin antibiotics, trypsin-EDTA (Sigma Aldrich, Singapore, Singapore) were used for culturing the cells. Alexa Fluor 647 Phalloidin (Life technologies Corporation Singapore, Singapore) and bovine serum albumin (Sigma-Aldrich, Singapore, Singapore) were used for analysing the protein expression. 1, 1, 1, 3, 3, 3-hexafluoro-2-propanol (HFIP), poly-ε-caprolactone, curcumin (CUR), tetracycline hydrochloride (TCH) (Sigma-Aldrich, Singapore, Singapore) and lyophilized Aloe vera (AV) powder (Xian Yuen Sun Biological Technology Co. Ltd., Shaanxi, China) to fabricate electrospun nanofibers. CellTiter 96^®^ and Cell Tracker Green CMFDA dye (Promega Pte. Ltd., Singapore, Singapore) and Sirius Red (Sigma-Aldrich, Singapore, Singapore). 

### 3.2. Fabrication of Electrospun Nanofibers

Polymer solution for electrospinning was prepared by dissolving PCL, AV, CUR, TCH in HFIP and stirred overnight at room temperature (RT). Five different combinations of PCL with natural polymers PCL (13%), PCL (10%)/AV (3%), PCL (10%)/CUR (3%), PCL (9%)/AV (3%)/CUR (1%), and PCL (9%)/AV (3%)/TCH (1%) were organized for electrospinning, with the ultimate concentration of 13% for all polymer composites. The polymer solution was loaded in a 5 mL standard syringe fitted with a capillary needle at a flow rate of approximately 1.5 mL/h controlled by a syringe pump (KDS 100, KD Scientific, Holliston, MA, USA) and high voltage 14–15 kV was applied by using high voltage power supply (Gamma High Voltage Research Inc., Ormond Beach, FL, USA) for electrospinning the nanofibers (Table 3). 

### 3.3. Characterization of Nanofibrous Scaffolds 

The electrospun nanofibers were sputter coated with gold JFC-1600 auto fine coater (JEOL, Peabody, MA, USA) and visualized using a field emission scanning electron microscope JSM-6701F FESEM (JEOL, Peabody, MA, USA). The diameters of the electrospun nanofibers were analyzed using image analysis software ImageJ Software (National Institutes of Health, Bethesda, MD, USA). The tensile properties of electrospun nanofibrous scaffolds were determined with a table-top tensile tester (Instron 3345, Instron Inc., Norwood, Ma, USA) using a load cell of 10 N capacity. The rectangular nanofiber mats of dimensions 10 mm width and 20 mm height were used for testing, at a crosshead speed of 10 mm/min and the data was recorded for every 50 s. The tensile stress, strain and elastic modulus were calculated based on the generated tensile stress-strain curve. The wettability of the electrospun nanofibrous scaffolds was measured by a sessile drop water contact angle measurement VCA optima surface analysis system (AST products, Billerica, MA, USA). The contact angle results reflected the hydrophilicity of the nanofibrous scaffolds. FTIR spectroscopic analysis of electrospun nanofibrous scaffolds was performed on (Bruker GmbH, Ettlingen, Germany) over a range of 500–4,000 cm^−1^ at a resolution of 2 cm^−1^.

### 3.4. Drug Release 

The release profile of CUR and TCH were analyzed by immersing the fabricated PCL/AV/CUR, PCL/AV/TCH nanofibrous mats in phosphate buffer saline (PBS) at 37 °C (pH 7.4). Approximately 50 mg of PCL/AV/CUR, PCL/AV/TCH mats were immersed in 3 mL of PBS solution and incubated at 37 °C. At time periods, 2 mL of PBS was withdrawn to study the release kinetics and substituted with the equal amount of fresh PBS. Then, 2 mL of PBS aliquot was further analyzed using a UV-Visible spectrophotometer at 420 nm for CUR and 350 nm for TCH. Using the calibration curve of CUR and TCH measured, the CUR and TCH release percentages were determined and plotted on the graph. 

### 3.5. Human Dermal Fibroblast (hDF) 

As primary human dermal fibroblast cells (hDF) play a key role in the wound healing process, hDF was used for determining the cytocompatibility of the electrospun mats. The hDF cells were cultured in DMEM medium (Gibco^®^, Thermo Fisher Scientific, Singapore, Singapore) supplemented with fetal bovine serum 10% (v/v), 50 µL mL^−1^ penicillin and 50 mg mL^−1^ streptomycin at 37 °C and 5% CO_2_ in a humidified incubator. The 15 mm scaffolds on the cover slips were sterilized under UV light for 3 h. Each of the nanofibrous scaffold on 15 mm cover slips was placed in a 24-well plate and round stainless-steel rings were added to each well to protect the fibers lifting from the coverslips. Then, each well with the scaffold was washed thrice with PBS and subsequently immersed in complete media overnight before cell seeding. For biocompatibility study experiments, the cells (1 × 10^4^ cells well^−1^) were seeded onto nanofiber collected coverslips, placed in 24-well plates and allowed to grow for 24 hrs before analysis. 

### 3.6. MTS Assay 

The cell proliferation was monitored on day 3, 6 and 9 by MTS (3-(4,5-dimethylthiazol-2-yl)-5-(3-carboxymethoxyphenyl)-2-(4-sulfophenyl)-2H-tetrazolium, inner salt) assay. The metabolically active cells react with tetrazolium salt in the MTS reagent to produce soluble formazan dye that can be observed at 490 nm. The cellular constructs were rinsed with PBS followed by incubation with 20% MTS reagent in serum free medium for 3 hrs. Thereafter, the aliquots were pipetted into 96 well plate and the samples were read in a spectrophotometric plate reader (FLUOstar OPTIMA, BMG Lab Technologies, Ortenberg, Germany) at 490 nm.

### 3.7. Cell-scaffold Interactions

The fibroblasts harvested on different scaffolds at day 9 were washed with PBS to remove non-adherent cells and then fixed in 4% glutaraldehyde for 1 hr at RT, dehydrated through a gradient of alcohol solution and finally, critical point dried using hexamethyldisilazane overnight to maintain the normal cell morphology. The dried cellular constructs were sputter coated with gold and observed under the scanning electron microscope at an accelerating voltage of 15 kV.

### 3.8. CMFDA Staining

On day 6, the scaffold-containing cells were washed with PBS after removing DMEM and stained with fluorescent molecules 5-Chloromethylfluorescein diacetate (CellTracker Green CMFDA, Promega, Singapore, Singapore). Labelling the cells was performed as described by the manufacturer. Briefly, the cells were incubated with dye at a concentration of 5 µM. After 1 hr of incubation at 37 °C, the CMFDA dye was discarded and the cells were washed with PBS. Futher the cells were incubated with the medium and supplemented with 10% fetal bovine serum at 37 °C for an additional 24 h to complete the labelling process. The cells were washed twice with PBS and mounted over the glass cover slides using the mounting medium. Fibroblast morphology was observed by a confocal laser scanning microscope at the wavelength of 490 nm.

### 3.9. Sirius Red Staining

Sirius red staining was used for analysing the presence of collagen in the cell secreted matrix. Sirius red is a strong anionic dye containing sulfonic acid groups, which interact with the basic group of collagen. On day 6, the cells were first fixed with 10% formaldehyde, stained with Harris’ haematoxylin to distinguish the nucleus of the cells and washed three times with deionized water. This was followed by staining with Sirius red stain consisting of 0.1% Sirius red F3B in a saturated aqueous solution of picric acid for 1 h. The cells were washed with mild acidified water followed by 100% ethanol and viewed under Leica DM IRB microscope. The collagen fibers were stained red on a yellow background.

### 3.10. F-actin Staining

The fibroblast cells cultured on the nanofibrous scaffolds were stained for F-actin on day 9 to analyze the expression of F-actin protein. The cells were first fixed in 100% ice-cold methanol for 15 min. The samples were washed with PBS for 15 min and incubated in Triton-X100 solution (0.5%) for 5 min which permeabilized the cell membrane. The non-specific binding sites were blocked by incubating the cells in 3% BSA for 1 h. The samples were then incubated with Alexa Fluor 647 Phalloidin in the dilution of 1:200 for 90 min. The samples were washed with PBS thrice to remove the excess staining and then incubated with DAPI in the dilution of 1:3000 for 30 min. The samples were then removed and mounted over a glass slide using a Vectashield mounting medium and examined under an Olympus FV1000 (USA) fluorescence microscope.

### 3.11. Disc Diffusion Assay

The antibacterial activity of electrospun mats were evaluated using the Kirby-Bauer radial disc diffusion method. The experiment was carried out in accordance with the Clinical and Laboratory Standards Institute (CLSI). Using a cotton swab, the bacterial cultures (concentration adjusted to 0.5 McFarland standards) were spread onto the sterile Muller Hinton Agar (MHA) plates. Electrospun mats (10 × 10 mm) were placed on the swabbed bacterial cultures and incubated at 35 ± 2 °C for 24 h. The antibacterial activity was assessed as a zone of inhibition in millimeters. The bacterial strains such as *Staphylococcus aureus*, *Staphylococcus epidermidis*, *Pseudomonas aeruginosa*, *Escherichia coli* and methicillin resistant *Staphylococcus aureus* (MRSA) were used for the study [45].

### 3.12. Statistical Analysis

The data presented were expressed as the mean ± standard deviation (SD). The statistical analysis was performed by one way analysis of variance (ANOVA), and significance was at *p* < 0.05.

## 4. Conclusions

The electrospinning technique used to fabricate TCH and CUR loaded PCL/AV nanofibrous antibacterial wound dressing mats for skin tissue engineering. The results proved that TCH loaded nanofibrous scaffolds are attributed with biocompatibility, good mechanical properties, hydrophilicity and antibacterial properties. The release study indicated an initial burst release followed by a sustained release of TCH. The release of TCH in the PCL/AV/TCH mat supported fibroblast growth, attachment, spreading along the nanofiber orientation with the enhanced deposition of collagen. The PCL/AV/TCH scaffold possessed a broad spectrum of antibacterial activity against both Gram-positive and Gram-negative bacteria. Overall, the TCH loaded PCL/AV mats displayed high biocompatibility, increased mechanical property, better surface wettability and antibacterial activity in comparison with PCL/AV mats loaded with CUR. Aloe vera is generally used for cosmetic applications, however, the authors are fabricating a modified AV membrane for diabetic wound healing applications. In conclusion, this study presents a smart scaffold with essential physical and biological properties that can topically deliver bioactive drugs to improve wound healing.

## Figures and Tables

**Table 1:** Fiber diameter and water contact angle of nanofibrous scaffolds. ** *p* < 0.01, **** *p* < 0.0001.

Nanofiber Construct	Fiber Diameter (nm)	Water Contact Angle (°)
PCL	770 ± 98	128.3 ± 6
PCL/AV	561 ± 49 ****	47.3 ± 2.5
PCL/CUR	695 ± 57 **	94.3 ± 3.7
PCL/AV/CUR	665 ± 64 ****	79 ± 1.6
PCL/AV/TCH	360 ± 87 ****	57.3 ± 5

**Table 2:** Mechanical properties of various electrospun nanofibrous scaffold.

Nanofiber Construct	Ultimate Tensile Stress (MPa)	Ultimate Tensile Strain (%)	Young’s Modulus (MPa)
PCL	9.2	164.5	19.8
PCL/AV	14.9	53.1	72.3
PCL/CUR	11.1	64.2	79.7
PCL/AV/CUR	14.4	65.2	25.5
PCL/AV/TCH	20.0	40.1	92.6

**Table 3:** Parameters applied for the fabrication of electrospun hybrid nanofibers.

Samples	Concentration (%)	Voltage (kV)	Flow Rate (ml/h)	Needle Size
PCL	13	14	1.5	24G
PCL/AV	10:3	14	1.5	24G
PCL/CUR	10:3	14	1.5	24G
PCL/AV/CUR	9:3:1	14	1.5	24G
PCL/AV/TCH	9:3:1	15	1.5	24G

## References

[1] Simpson C.L., Patel D.M., Green K.J. (2011). Deconstructing the skin: Cytoarchitectural determinants of epidermal morphogenesis. Nat. Rev. Mol. Cell Biol..

[2] Vig K., Chaudhari A., Tripathi S., Dixit S., Sahu R., Pillai S., Dennis V.A., Singh S.R. (2017). Advances in skin regeneration using tissue engineering. Int. J. Mol. Sci..

[3] Ozcelik H., Hindie M., Hasan A., Engin N., Cell V., Barthes J., Özçelik H., Hindié M., Ndreu-halili A., Vrana N.E. (2014). Cell microenvironment engineering and monitoring for tissue engineering and regenerative medicine. Biomed Res. Int..

[4] Olson J.L., Atala A., Yoo J.J. (2011). Tissue Engineering: Current Strategies and Future Directions. Chonnam Med. J..

[5] Inchingolo F., Tatullo M., Marrelli M., Inchingolo A.M., Inchingolo A.D., Dipalma G., Flace P., Girolamo F., Tarullo A., Laino L. (2012). Regenerative surgery performed with platelet-rich plasma used in sinus lift elevation before dental implant surgery: An useful aid in healing and regeneration of bone tissue. Eur. Rev. Med. Pharmacol. Sci..

[6] Nayak S., Dey S., Kundu S.C. (2013). Skin equivalent tissue-engineered construct: Co-cultured fibroblasts/ keratinocytes on 3D matrices of sericin hope cocoons. PLoS ONE.

[7] Chan B.P., Leong K.W. (2008). Scaffolding in tissue engineering: General approaches and tissue-specific considerations. Eur. Spine J..

[8] Paduano F., Marrelli M., Alom N., Amer M., White L.J., Shakesheff K.M., Tatullo M. (2017). Decellularized bone extracellular matrix and human dental pulp stem cells as a construct for bone regeneration. J. Biomater. Sci. Polym. Ed..

[9] Strong A.L., Neumeister M.W., Levi B. (2018). Stem cells and tissue engineering: Regeneration of the skin and its contents. Clin Plast Surg..

[10] Andreu V., Mendoza G., Arruebo M., Irusta S. (2015). Smart dressings based on nanostructured fibers containing natural origin antimicrobial, anti-inflammatory, and regenerative compounds. Materials (Basel).

[11] Harrison K. (2007). Introduction to polymeric scaffolds for tissue engineering. Biomedical Polymers.

[12] Kim P.H., Cho J.Y. (2016). Myocardial tissue engineering using electrospun nanofiber composites. BMB Rep..

[13] Agarwal S., Wendorff J.H., Greiner A. (2009). Progress in the field of electrospinning for tissue engineering applications. Adv. Mater..

[14] Ulery B.D., Nair L.S., Laurencin C.T. (2011). Biomedical applications of biodegradable polymers. J. Polym. Sci. Part B Polym. Phys..

[15] Khosravi A., Ghasemi-Mobarakeh L., Mollahosseini H., Ajalloueian F., Masoudi Rad M., Norouzi M.R., Sami Jokandan M., Khoddami A., Chronakis I.S. (2018). Immobilization of silk fibroin on the surface of PCL nanofibrous scaffolds for tissue engineering applications. J. Appl. Polym. Sci..

[16] Amar S., Resham V., Saple D.G. (2008). Aloe Vera: A Short Review. Indian J. Dermatol..

[17] Rahman S., Carter P., Bhattarai N. (2017). Aloe Vera for Tissue Engineering Applications. J. Funct. Biomater..

[18] Kant V., Gopal A., Pathak N.N., Kumar P., Tandan S.K., Kumar D. (2014). Antioxidant and anti-inflammatory potential of curcumin accelerated the cutaneous wound healing in streptozotocin-induced diabetic rats. Int. Immunopharmacol..

[19] Zorofchian Moghadamtousi S., Abdul Kadir H., Hassandarvish P., Tajik H., Abubakar S., Zandi K. (2014). A review on antibacterial, antiviral, and antifungal activity of curcumin. Biomed Res. Int..

[20] Garrido-Mesa N., Zarzuelo A., Gálvez J. (2013). Minocycline: Far beyond an antibiotic. Br. J. Pharmacol..

[21] Soares P.B.F., de Menezes H.H.M.H., de Naves M.M., Taga E.M., de Magalhães D. (2009). Effect of absorbent tetracycline-loaded membrane used in the reduction of periodontal pockets: An in vivo study. Braz. Dent. J..

[22] Agnes Mary S., Giri Dev V.R. (2015). Electrospun herbal nanofibrous wound dressings for skin tissue engineering. J. Text. Inst..

[23] Lee D.E., Ayoub N., Agrawal D.K. (2016). Mesenchymal stem cells and cutaneous wound healing: Novel methods to increase cell delivery and therapeutic efficacy. Stem Cell Res. Ther..

[24] Li P., Guo X. (2018). A review: Therapeutic potential of adipose-derived stem cells in cutaneous wound healing and regeneration. Stem Cell Res. Ther..

[25] Parisi L., Toffoli A., Ghiacci G., Macaluso G.M. (2018). Tailoring the interface of biomaterials to design effective scaffolds. J. Funct. Biomater..

[26] Karuppuswamy P., Venugopal J.R., Navaneethan B., Laiva A.L., Sridhar S., Ramakrishna S. (2014). Functionalized hybrid nanofibers to mimic native ECM for tissue engineering applications. Appl. Surf. Sci..

[27] Shababdoust A., Ehsani M., Shokrollahi P., Zandi M. (2017). Fabrication of curcumin-loaded electrospun nanofiberous polyurethanes with anti-bacterial activity. Prog. Biomater..

[28] Širc J., Hobzová R., Kostina N., Munzarová M., Juklíčková M., Lhotka M., Kubinová Š., Zajícová A., Michálek J. (2012). Morphological characterization of nanofibers: Methods and application in practice. J. Nanomater..

[29] Balaji A., Jaganathan S.K., Ismail A.F., Rajasekar R. (2016). Fabrication and hemocompatibility assessment of novel polyurethane-based bio-nanofibrous dressing loaded with honey and Carica papaya extract for the management of burn injuries. Int. J. Nanomedicine.

[30] Shah S.A.A., Imran M., Lian Q., Shehzad F.K., Athir N., Zhang J., Cheng J. (2018). Curcumin incorporated polyurethane urea elastomers with tunable thermo-mechanical properties. React. Funct. Polym..

[31] Trang Mai T.T., Thuy Nguyen T.T., Le Q.D., Nguyen T.N., Ba T.C., Nguyen H.B., Hoa Phan T.B., Tran D.L., Nguyen X.P., Park J.S. (2012). A novel nanofiber Cur-loaded polylactic acid constructed by electrospinning. Adv. Nat. Sci. Nanosci. Nanotechnol..

[32] Liao N., Unnithan A.R., Joshi M.K., Tiwari A.P., Hong S.T., Park C.H., Kim C.S. (2015). Electrospun bioactive poly (ε-caprolactone)–cellulose acetate–dextran antibacterial composite mats for wound dressing applications. Colloids Surfaces A Physicochem. Eng. Asp..

[33] Suganya S., Venugopal J., Agnes Mary S., Ramakrishna S., Lakshmi B.S., Giri Dev V.R. (2014). Aloe vera incorporated biomimetic nanofibrous scaffold: A regenerative approach for skin tissue engineering. Iran. Polym. J..

[34] Lee H., Yamaguchi K., Nagaishi T., Murai M., Kim M., Wei K., Zhang K.Q., Kim I.S. (2017). Enhancement of mechanical properties of polymeric nanofibers by controlling crystallization behavior using a simple freezing/thawing process. RSC Adv..

[35] Xiang C., Frey M.W. (2016). Increasing mechanical properties of 2-D-structured electrospun nylon 6 non-woven fiber mats. Materials (Basel).

[36] Wongkanya R., Chuysinuan P., Pengsuk C., Techasakul S., Lirdprapamongkol K., Svasti J., Nooeaid P. (2017). Electrospinning of alginate/soy protein isolated nanofibers and their release characteristics for biomedical applications. J. Sci. Adv. Mater. Devices.

[37] Chen S.C., Huang X.B., Cai X.M., Lu J., Yuan J., Shen J. (2012). The influence of fiber diameter of electrospun poly(lactic acid) on drug delivery. Fibers Polym..

[38] Kumar S., Yadav A., Yadav M., Yadav J.P. (2017). Effect of climate change on phytochemical diversity, total phenolic content and in vitro antioxidant activity of Aloe vera (L.) Burm.f. BMC Res. Notes.

[39] Xue M., Jackson C.J. (2015). Extracellular matrix reorganization during wound healing and its impact on abnormal scarring. Adv. Wound Care.

[40] Wakuda Y., Nishimoto S., Suye S.I., Fujita S. (2018). Native collagen hydrogel nanofibres with anisotropic structure using core-shell electrospinning. Sci. Rep..

[41] Brett D. (2008). A review of collagen and collagen-based wound dressings. Wounds.

[42] Jin G., Prabhakaran M.P., Kai D., Annamalai S.K., Arunachalam K.D., Ramakrishna S. (2013). Tissue engineered plant extracts as nanofibrous wound dressing. Biomaterials.

[43] Abreu-Blanco M.T., Watts J.J., Verboon J.M., Parkhurst S.M. (2012). Cytoskeleton responses in wound repair. Cell. Mol. Life Sci..

[44] Delaine-Smith R.M., Green N.H., Matcher S.J., MacNeil S., Reilly G.C. (2014). Monitoring fibrous scaffold guidance of three-dimensional collagen organisation using minimally-invasive second harmonic generation. PLoS ONE.

[45] Ramalingam R., Dhand C., Leung C.M., Ezhilarasu H., Prasannan P., Ong S.T., Subramanian S., Kamruddin M., Lakshminarayanan R., Ramakrishna S. (2019). Poly-ε-caprolactone/gelatin hybrid electrospun composite nanofibrous mats containing ultrasound assisted herbal extract: Antimicrobial and cell proliferation study. Nanomaterials.

